# Value of foetal umbilical vein standardised blood flow volume in predicting weight gain in the third trimester: a prospective case-cohort study

**DOI:** 10.3389/fped.2024.1376774

**Published:** 2024-07-16

**Authors:** Qian Fang, Yihao Shi, Chao Zhang, Ying Cai, Cuili Yuan, Jiaxiang Yang, Guannan He

**Affiliations:** ^1^ Department of Ultrasound, LongQuanYi District of Chengdu Maternity and Child Health Care Hospital, Chengdu, China; ^2^ Department of Statistics, LongQuanYi District of Chengdu Maternity and Child Health Care Hospital, Chengdu, China; ^3^ Department of Ultrasound, Sichuan Provincial Maternity and Child Health Care Hospital, Chengdu, China

**Keywords:** foetus, umbilical vein, blood flow, standardised blood flow volume, body weight

## Abstract

**Objective:**

This study aims to establish a prediction model of foetal umbilical vein standardised blood flow volume (sQuv) on estimated foetal weight (EFW) in the third trimester.

**Methods:**

A case-cohort study involving 200 eligible normal foetuses was conducted at the Ultrasound Department of Longquanyi District of Maternity and Child Healthcare Hospital between June 1, 2020 and December 31, 2021. Ultrasound measurements were taken at two separate intervals to assess EFW and the rate of EFW (rEFW) [first: between 28 w and 33 w6d of gestational age (GA); second: after 4–6 weeks]. Umbilical vein blood flow volume (Quv) and sQuv (normalised with EFW) were calculated only during the initial measurement. Using general linear regression, a prediction model for EFW based on GA and sQuv was developed, with the gestational week employed as a calibration scalar and validated using linear regression cross-validation.

**Results:**

In the third trimester, EFW exhibited significant correlations with GA, abdominal circumference (AC), head circumference (HC) and Quv (all *ρ* > 0.6, *P* < 0.001). Furthermore, the rEFW showed significant correlations with Quv and sQuv (all *ρ* > 0.6, *P* < 0.001). A linear regression equation was established using a general linear regression model: rEFW = 0.32689 × sQuv. Additionally, a foetal weight prediction model (EFW = −2,554.6770 + 0.9655 × sQuv + 129.6916 × GA) was established using sQuv. The above two formulas were cross-validated by intra-group linear regression and proved to be of good efficacy.

**Conclusions:**

In the third trimester, EFW displayed significant correlations with GA, AC, HC and Quv. Additionally, the rEFW exhibited significant correlations with Quv and sQuv. The sQuv during the third trimester has predictive value for foetal weight, serving as an early warning indicator.

## 1 Introduction

Foetal weight, as the paramount indicator of intrauterine growth and development, significantly influences the condition of the foetus after birth. Birth weight is closely associated with various perinatal complications: intrauterine growth restriction may lead to foetal hypoxia, neonatal asphyxia, meconium aspiration syndrome, etc. (1). Macrosomic pregnancies may result in obstructed labour, such as prolonged labour or postpartum haemorrhage (2, 3). Additionally, research suggests that intrauterine overgrowth may be linked to long-term adverse outcomes in children, including an increased risk of hypertension and type 2 diabetes (4, 5).

Traditional methods of assessing foetal weight involve measuring maternal weight, abdominal circumference (AC) and fundal height. However, ultrasound has revolutionised this process, overcoming the limitations of traditional measurements. Foetal growth is closely related to placental circulation, with the foetal umbilical vein serving as the origin of foetal blood circulation (6–8). The umbilical vein is the sole pathway through which the foetus receives blood and nutrients from the mother. Sinkovskaya et al. (9) conducted a comprehensive observation and investigation of the foetal vein across six anatomical levels using ultrasound instruments. Initially, early studies predominantly focused on the poor prognosis of the foetus resulting from anatomical abnormalities of the umbilical vein (10). However, over time, more research turned towards foetal umbilical vein blood flow, making it a focal point of maternal–foetal medicine research.

This prospective study selected normal foetuses in the third trimester as an observational cohort. Ultrasound techniques were employed to measure and calculate foetal umbilical vein blood flow parameters to analyse the correlation between umbilical vein blood flow and foetal weight in the third trimester, as well as the distribution of blood flow. By exploring the predictive value of standardised blood flow volume on body weight, this study aims to provide obstetricians with a more effective reference index for assessing and predicting foetal body weight.

## 2 Materials and methods

### 2.1 Study population

Two hundred eligible foetuses were selected from pregnant patients who underwent ultrasound examinations at the Ultrasound Department of Longquanyi District of Chengdu Maternity and Child Health Care Hospital between June 1, 2020 and December 31, 2021. This study was conducted in accordance with the Declaration of Helsinki and approved by the ethics committee of Longquanyi District of Chengdu Maternity and Child Health Care Hospital (KY-2020-04). Written informed consent was obtained from all participants.

The inclusion criteria were as follows: (1) foetuses of healthy pregnant women aged 20–34 years who voluntarily signed the Informed Consent for Research; (2) singleton pregnancies normally delivered at full term (38–42 weeks). The exclusion criteria were as follows: (1) pregnant women with hypertension, diabetes mellitus, cardiorespiratory abnormalities or history of hereditary diseases; (2) foetuses with structural malformations, chromosomal abnormalities, hydrops fetalis, oligohydramnios, umbilical artery abnormalities, right umbilical vein, portosystemic shunt, cord knots or cord entanglement; (3) foetuses clinically diagnosed as growth-restricted, small for gestational age (SGA) or large for GA (LGA).

### 2.2 Design of case-cohort study

A study cohort comprising eligible foetuses underwent two consecutive ultrasound examinations in the third trimester. The first examination was performed between GA of 28 w and 33 w6d, during which the weight and umbilical vein blood flow volume (Quv) were measured. The second examination was carried out 4–6 weeks later (GA’), during which estimated foetal weight (EFW) and the rate of EFW (rEFW) between the two intervals were calculated. Foetal GA was determined based on early gestational nuchal translucency examination (estimated GA derived from head and hip length measured by ultrasound).

### 2.3 Equipment and measurement

A GE-E8 colour ultrasonic diagnostic instrument equipped with a transabdominal volumetric probe (model C1–5) operating at a frequency of 1–5 MHz was used (safety description: spatial peak time-averaged sound intensity <100 mW/cm^2^, mechanical variation of 10%). Initially, two attending physicians, each with over 5 years of experience in obstetric ultrasound examinations, underwent certification and quality assessment. Subsequently, procedures for image acquisition, data measurement and image storage were carried out (from 14:00 to 16:00 every day).

Measurements of foetal growth parameters, such as biparietal diameter, head circumference (HC), AC and femur length, were conducted thrice, and the mean value was computed. Based on the above parameters, the ultrasonic instrument's software employed Hadlock's formula to calculate ultrasound EFW in grams (11).

**Figure 1:**
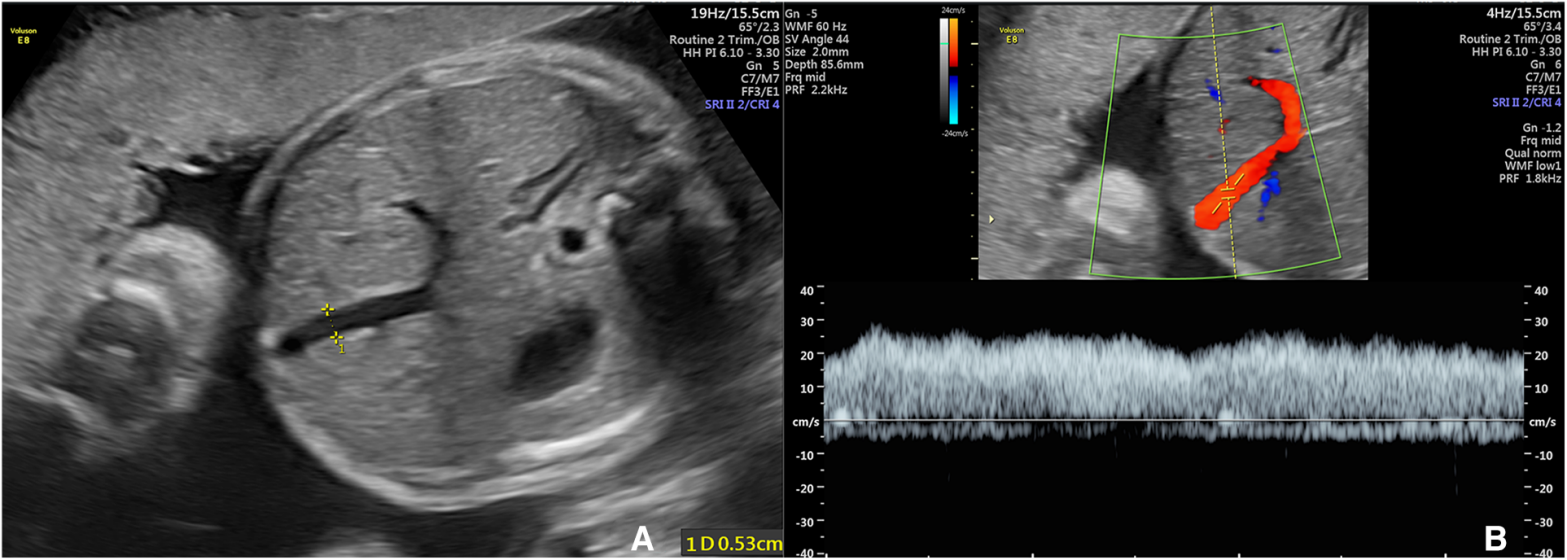
The ultrasonic measurment of fetal umbilical vein. (**A**) Velocity of umbilical venous blood flow (Vuv); (**B**) Diameter of umbilical venous blood flow (Duv).

Umbilical vein measurements were made by deflecting the transverse section of the foetal abdominal circumference slightly towards the entrance of the abdominal wall of the umbilical cord (Figure 1). The procedure involved the following. (1) The morphology of the intra-abdominal segment of the umbilical vein was displayed using colour Doppler flow imaging mode. (2) The umbilical vein spectrum was displayed using pulsed-wave Doppler mode: a sample volume of 0.1–0.2 cm was placed approximately 1.0 cm into the abdomen within the umbilical vein, ensuring the sound velocity was parallel to blood flow and the angle correction was <30°. More than three complete waveforms were consecutively displayed, followed by mean velocity (Vuv) measurements. (3) Using the Zoom function of two-dimensional ultrasound imaging (B-Mod) mode to locally magnify the same position and measure the inner diameter of the umbilical vein (Duv). (4) Sampling was conducted during periods of foetal inactivity when the patient could suspend breathing. Measurements were repeated three times, and the mean values were recorded (12, 13).

### 2.4 Data operation

The Quv was calculated using the following formula (12): Quv (ml/min) = π × [Duv (cm)/2]^2 ^× Vuv (cm/s) × 60 (s/min).

The umbilical vein standardised blood flow volume (sQuv) was calculated using the following formula (13): sQuv (ml/min/kg) = Quv (ml/min)/EFW (g) × 1,000.

The rate of foetal weight gain was determined by two GA points and their corresponding EFW. The rEFW was calculated using the following formula: rEFW (g/w) = [EFW’ (g)−EFW (g)]/[GA’ (w)−GA (w)].

### 2.5 Statistical analysis

All statistical analyses were performed using SPSS software (version 27.0, IBM Corp., USA) and R 4.2.2 (R Core Foundation, Austria). This study developed a linear regression prediction model by employing the lm function in the R language and cross-validated its formula against the cv.glm function in the boot package (k = 10). Quantitative data following a normal distribution were presented as mean ± standard deviation (x ± s), and non-normally distributed quantitative data were depicted using the interquartile range (25%–75%). Count data were expressed as cases (*n*) and percentages (%). Pearson's (normal distribution) or Spearman's (non-normal distribution) correlation analysis was performed. Regression analysis and linear fitting were conducted for variables with significant correlation at the level of *P *< 0.001, and the general linear model was employed to fit the optimal curve to establish the prediction model. Furthermore, within-group cross-validation was conducted to assess the effectiveness of the model.

## 3 Results

The umbilical vein flow and growth trajectories of 200 foetuses in the third trimester were measured and recorded using ultrasound instruments (Table 1). Concerning foetal weight, the mean value of EFW in the third trimester was obtained as 1,161 g (1,183–2,218 g), and the mean value of the rEFW in the third trimester was 185.2 g/w (110–278.4 g/w). For umbilical vein blood flow, the mean value of Quv was obtained as 288.9 ml/min (90–598 ml/min) in the third trimester. This study standardised the blood flow volume based on the foetal weight and yielded a mean value of 176 ml/min/kg (67.6–323.4 ml/min/kg) for the standardised blood flow volume (sQuv).

**Table 1:** Baseline characteristics of fetal EFW and umbilical blood flow.

*N* = 200	Maximum value	Minimum value	Mean value	Standard deviation
GA(weeks)	28.6	33.4	30.8	0.909
EFW (g)	1,183	2,218	1,611	0.094
HC (cm)	26.3	31.5	28.5	1.013
AC (cm)	23.6	29.7	26.3	1.195

Spearman's correlation analysis was employed to calculate the correlation coefficient between umbilical blood flow parameters and body weight parameters. The correlation coefficients were presented using a matrix (Table 2). In the third trimester, EFW exhibited significant correlations with the GA (*ρ* = 0.681, *P *< 0.001), AC (*ρ* = 0.949, *P *< 0.001), HC (*ρ* = 0.792, *P *< 0.001) and Quv (*ρ* = 0.638, *P *< 0.001). Furthermore, the rEFW in the third trimester showed significant correlations with Quv (*ρ* = 0.628, *P *< 0.001) and sQuv (*ρ* = 0.641, *P *< 0.001).

**Table 2:** Spearman correlation analysis and correlation matrix.

	EFW	rEFW	GA	HC	AC	Duv	Vuv	Quv	sQuv
EFW	1.000	0.310	0.681	0.792	0.949	0.645	0.146	0.638	0.440
*P* value		<.0001	<.0001	<.0001	<.0001	<.0001	0.039	<.0001	<.0001
rEFW		1.000	0.265	0.314	0.295	0.346	0.407	0.628	0.641
*P* value			0.000	<.0001	<.0001	<.0001	<.0001	<.0001	<.0001
GA			1.000	0.667	0.616	0.468	0.078	0.433	0.299
*P* value				<.0001	<.0001	<.0001	0.274	<.0001	<.0001
HC				1.000	0.705	0.496	0.215	0.568	0.422
*P* value					<.0001	<.0001	0.002	<.0001	<.0001
AC					1.000	0.606	0.141	0.606	0.417
*P* value						<.0001	0.047	<.0001	<.0001
Duv						1.000	−0.237	0.533	0.418
*P* value							0.001	<.0001	<.0001
Vuv							1.000	0.646	0.719
*P* value								<.0001	<.0001
Quv								1.000	0.966
*P* value									<.0001
sQuv									1

**Figure 2:**
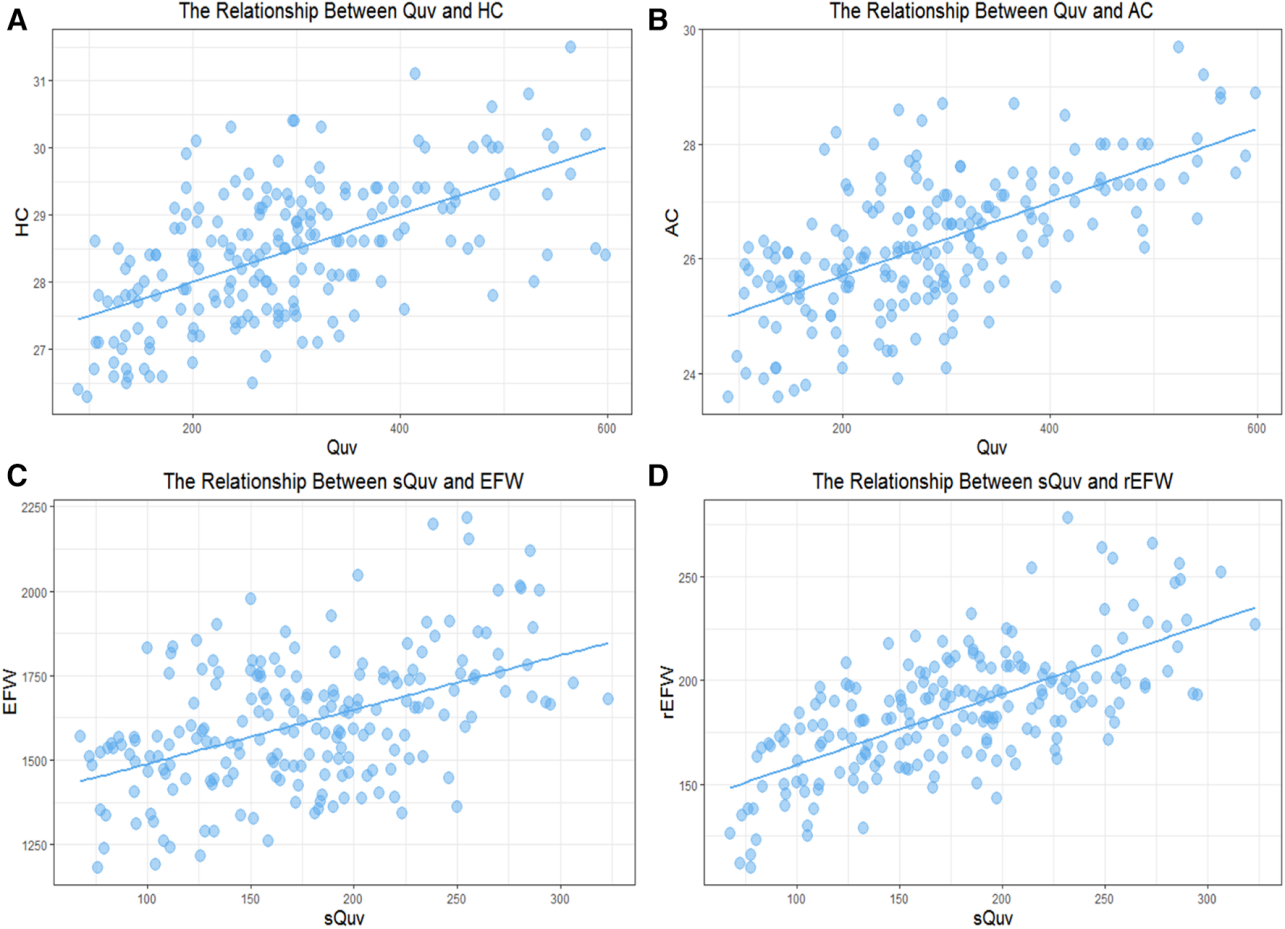
The relationship between fetal umbilical vein blood flow and weight parameters. (**A**) The relationship between Quv and HC; (**B**) The relationship between Quv and AC; (**C**) The relationship between sQuv and EFW. (**D**) The relationship between sQuv and rEFW.

Differential correlations of foetal Quv with HC, as well as Quv with AC, were found. This distinction was illustrated through scatterplots and regression lines (Figures 2A,B).

Finally, this study standardised the Quv based on foetal weight to yield the sQuv. The correlation between the sQuv and the rEFW was demonstrated using a scatter plot and a regression line, respectively (Figure 2C). As a result, a linear regression formula was established for the sQuv and the rEFW: rEFW = 0.32689 × sQuv (Table 3), which was validated as having good efficacy through within-group cross-validation (Table 4).

**Table 3:** Regression analysis of independent variable sQuv and dependent variable rEFW.

	Estimate	Std. Error	*t* value	Pr (>|t|)
(Intercept)	47.86889	53.914	0.888	0.376
sQuv	0.32689	0.028	11.672	<0.001
GA	2.58891	1.793	1.444	0.150

R-squared: 0.4565, Adjusted R-squared: 0.451. Linear regression equation of independent variable sQuv and dependent variable rEFW: rEFW = 0.32689 × sQuv.

**Table 4:** Cross-validation for generalized linear models.

k	MSE	RMSE
1	485.199	22.027
2	485.118	22.025
3	485.004	22.023
4	485.653	22.038
5	480.924	21.930
6	489.539	22.126
7	489.480	22.124
8	486.907	22.066
9	480.701	21.925
10	483.575	21.990

rEFW, 0.32689 × sQuv.

Following the linear regression formula of the sQuv and the rEFW, the prediction formula of sQuv for EFW was established in the third trimester: EFW = −2,554.6770 + 0.9655 × sQuv + 129.6916 × GA (Table 5), which was validated as having good efficacy by within-group cross-validation (Table 6). Scatter plots and regression lines were used to demonstrate the high correlation between the sQuv and the rEFW, respectively (Figure 2D).

**Table 5:** Correction of sQuv based on GA.

	Estimate	Std.Error	*t* value	Pr(>|t|)
(Intercept)	−2,554.6770	321.0256	−7.958	<0.001
sQuv	0.9655	0.1668	5.79	<0.001
GA	129.6916	10.6774	12.146	<0.001

R-squared: 0.5598, Adjusted R-squared: 0.5553. Linear regression equation of independent variable sQuv and dependent variable EFW: EFW = −2,554.6770 + 0.9655 × sQuv + 129.6916 × GA.

**Table 6:** Cross-validation for generalized linear models.

k	MSE	RMSE
1	17,106.10	130.790
2	16,998.54	130.378
3	17,092.56	130.739
4	17,278.64	131.448
5	17,272.82	131.426
6	17,354.60	131.737
7	17,227.51	131.254
8	17,016.90	130.449
9	17,013.10	130.434
10	17,279.02	131.450

EFW, −2,554.6770 + 0.9655 × sQuv + 129.6916 × GA.

## 4 Discussion

This study established a cohort of 200 patients exhibiting normal foetal weight gain in the third trimester, and ultrasound techniques were applied to measure foetal Quv. Upon confirming a significant correlation between foetal Quv and EFW, this study developed a linear regression equation incorporating a novel metric (sQuv) with the rEFW. Subsequently, a predictive model for EFW was established for foetal GA and sQuv, which demonstrated positive validation results for its efficacy. This model has the potential to serve as an early warning system for foetuses at risk of abnormal weight gain during the third trimester. The findings of this prospective study can enhance the management of foetal weight during this crucial period.

In recent years, there have been significant advancements in ultrasound instrumentation technology, which have greatly improved the visualisation and measurement of Quv, providing a more comprehensive understanding of the foetal umbilical venous system. Among the limited studies on the umbilical vein of normal foetuses, investigations into the relationship between umbilical vein diameter and GA, flow velocity and GA and flow volume and GA have been conducted (14, 15). Foetal umbilical vein diameter and flow velocity showed a significant positive correlation with GA over a longer period from 20 to 40 weeks of GA (16–18). This study opted to only include foetuses in the third trimester due to the large variation in foetal weight and rapid rate of weight gain at the same GA during this period. These findings demonstrated that the correlation between Quv and body weight was more pronounced than that between blood flow volume and GA in foetuses in late pregnancy.

Over the past two decades, advancements in early ultrasound technology have facilitated precise and consistent measurements of the fetal umbilical artery. Consequently, findings from numerous studies regarding the relationship between the fetal umbilical artery and body weight have been extensively incorporated into obstetric management (19). For example, the research conducted by Angelo Sirico et al. elegantly elucidates the correlation between the umbilical artery pulsatility index and the estimated fetal weight percentile and birthweight percentile (20–22).With recent technological improvements in ultrasound instrumentation for venous measurements, obstetricians anticipate further insights into the correlation between the foetal umbilical vein and body weight, which could significantly inform obstetric practices (23). Of the few studies that have addressed foetal umbilical vein and weight, in most cases, foetuses with abnormal weight development were selected for clinical control studies. These studies primarily explored three key areas as follows. (1) First was the diagnostic value of foetal Quv in identifying intrauterine growth restriction (24–26). Among these, a meta-analysis highlighted the umbilical vein's ability to distinguish between early-onset and late-onset foetal growth restriction (19). (2) Second, researchers such as Fatihoglu and Parra-Saavedra used umbilical vein flow to predict the occurrence of SGA (27, 28). (3) In the third trimester of pregnancy, the predictive value of foetal umbilical vein blood flow parameters for LGA has also received obstetric attention (29, 30). This study took a different perspective, focusing primarily on the relationship between Quv and foetal weight, as well as weight gain, in normally developing foetuses during late pregnancy. It is hoped that these findings will contribute to monitoring the normal growth and development of foetuses.

Measurements of foetal Quv can be interfered with by maternal respiration and intrauterine foetal activity, necessitating stringent quality control for testing personnel (31). However, blood flow volume can more directly reflect the blood supply of the foetus. In recent years, the concept of standardised blood flow rate has been introduced on top of blood flow volume to show the average distribution of blood flow per unit of body weight, making the differences in blood flow in the bodies of foetuses of different sizes more apparent (3). In this study cohort, Spearman's correlation coefficient between foetal Quv and EFW was 0.638, and that between sQuv and the rEFW was 0.641, neither of which correlated extremely well above 0.8. It is believed that this finding may better reflect the objective relationship between foetal Quv and growth in the real world. Blood flow volume represents the physical aspect of foetal blood access. However, various biochemical substances in the blood play crucial roles as biological and chemical factors for foetal blood and nutrient acquisition, including vascular growth factors and blood sugar (32, 33). Scholars from different countries have investigated the biochemical substances in foetal umbilical vein blood flow and concluded that multiple biochemical substances jointly affect foetal weight gain (34).

Nevertheless, there are still some limitations in this study. Firstly, the samples included were confined to a single hospital, whereas the predictive model would benefit from validation on foetal samples from other healthcare facilities. Secondly, as a longitudinal observation in a cohort study, the duration of this study was limited to the third trimester. In future studies, this could be extended to the second trimester to broaden the application of the model. Thirdly, this study only investigated blood flow volume in the umbilical vein, overlooking the influence of factors in the umbilical artery and significant biochemicals in the blood, potentially constraining the generalizability of the results. The correlation between foetal Quv and body weight in the real world may involve numerous factors of haemodynamics, biology and chemistry. More comprehensive research information can only be obtained through increased multidisciplinary collaboration.

In summary, this study provides valuable insights into predicting the growth trend of normal foetuses in the third trimester, emphasising its potential implications for both research and practical applications. In the third trimester, EFW displayed significant correlations with GA, AC, HC and Quv. Additionally, the rEFW exhibited significant correlations with Quv and sQuv. Using the predictive model, obstetricians can proactively identify foetal weight abnormalities during this critical development phase. Based on the findings of this study, there is significant potential for further investigation into modulating foetal Quv, thereby optimising nutrient delivery to the foetus. Innovative and efficient explorations within this field are eagerly anticipated to be embarked upon in the future.

## Data Availability

The original contributions presented in the study are included in the article/Supplementary Material, further inquiries can be directed to the corresponding author.
